# Disease of the Gum Borders and Sockets of the Teeth

**Published:** 1901-09-15

**Authors:** C. M. Wright

**Affiliations:** Cincinnati


### DISEASE OF THE GUM BORDERS AND SOCKETS OF THE TEETH.

BY C. M. WRIGHT, B.S., A.M., D.D.S., CINCINNATI.

Abstract of paper read before the Tri-State Dental Meeting, Indianapolis, Ind., June 6, 1901.

This subject has been referred to in medical literature for hundreds of years, and for the past quarter of a century has been the special study of many of the brightest minds in our profession. To-day it has a literature so full, a nomenclature so rich, an aspect so apparent, and yet possible causes so obscure, that the profession and the public are fascinated by the mystery that seems to be bound up in this small section of tissue defined by a narrow border of gum and the so-called sockets of the teeth. The dentist, in making an examination of the teeth, instead of simply counting the number of cavities, critically examines the gums about the necks of the teeth for evidences of a tendency toward, or the actual presence of, a condition which is so prevalent that some authorities claim that 60 percent of the teeth of all persons over thirty years of age are lost by it. A few modern dentists devote a large proportion of their time and practice to the treatment of this one disease.

A quarter of a century ago little attention was given to this condition by the great body of dentists, further than to extract loose teeth and those surrounded by spongy and bleeding gums. Enucleation was a surgical operation of considerable dignity with the dentist at that time. It was largely employed for "ulcerated teeth," for persistent fistulæ from the roots, and by many even for molars and bicuspids with "exposed and painful nerves."

Riggs' disease, *alias* pyorrhea alveolaris, *alias* phagedenic pericementitis, interstitial gingivitis, utitis purulosis, pericemental necrobiosis and other histological and pathological *aliases* have been passing through all the fluctuations of theoretical and experimental eruptions; yet its etiology is really no more obscure than that of catarrh of the respiratory passages. In therapeutics, the general practitioner, as well as the specialists in rhinology and larnygology, find just as good a field in catarrh for continued treatment and permanent income (Whittaker) as the dentist can in this disease about which we are writing. In catarrh the causes are undoubtedly both local and constitutional. If the causes were purely local the disease would be limited to one or two recognized phases or stages, like hyperemia with its bland secretions, or simple acute inflammation with the more strenuous efforts on the part of the blood and tissues in the direction of regeneration, and with no tendency toward recurrence after the local irritant had been removed.

In this disease the circulation of the part is disordered, and we have in many cases an active and afterward a passive hyperemia or congestion; simultaneously we have exudates from the blood, which vary according to the progress of the disease and changes in the circulation and tissues. A serous secretion at first may later be fibrinous, hemorrhagic, muco-purulent, purulent. The tissues in this circumscribed area are soaked with an excess of blood exudate, and take on disordered nutritive action, become reduced to conditions resembling embryonic tissue; but the cellular elements multiply and try to organize and rebuild. Irritants from without, mechanical and chemical, and obstructions and toxines from within oppose the struggle for existence on the part of the protoplasm of the cells, and we have the complications of a *chronic* disease. At any stage of the conflict the dental surgeon can afford aid. With his specially educated touch, his well-tempered instruments, selected to suit his own hand, and with disinfectants and other agents that promote waste or stimulate and encourage repair, he is well equipped to battle with local irritants and aid the efforts of the tissues.

When we reflect upon the methods the tissues themselves display in efforts toward a physiological type, dealing with the exudates and necrosed tissue by absorption or sequestration and expulsion, and in the meantime by granulation and regenerative methods struggling to repair and restore to health, we can understand that the more perfect our recognition of the exact pathological state at any given period of the disease, the better able shall we be to assist rather than hinder these efforts on the part of the tissues themselves. Therefore, *judgment* is as important as surgical dexterity and thoroughness on the part of the operator.

To lay out for your inspection a lot of instruments, or to define when to use iodine, hot water, or trichoracetic acid, or any other remedies, is not the business of this paper, though it is the business of the surgeon to know about them. Simply to indicate clearly the relation this disease bears to others, to classify it, to define its position in pathology, in therapeutics, in dentistry, and, if I may say, in comparative specialism, is all that falls to our province.

The disease generally presents catarrhal, interstitial and phagedenic inflammatory phases, because of the kinds of epithelial and connective tissues involved and because of the location and unique characteristics of the arrangement and interdependence of these tissues.

Histology shows us that the pericemental connective tissue in early life is less dense and fibrous, and more myxotomous than in middle life and old age; that these peridental membranes are prone to senile changes and degenerations, liable to obsolescence; that there is probably a glandular excretive function of the epithelia just under the free border of the gum.

With such transitional structures, so functionally complex, and in such exposed situations, inflammations from causes ectogenous, and these innumerable, as we can readily see, such as calcareous deposits, food debris, bacterial activity, traumatic injuries, tooth-brush bristles, and a host of other things, or causes hematogenous or lymphogenous, such as waste products in the plasma that have exuded from the capillary loops of the parts and have not been carried away by the lymph. Any of us can picture the irritation that might produce inflammation in these gum borders from a disabled kidney, or a torpid liver, or from auto-infection from the alimentary canal, or from nerve exhaustion ; all manifesting themselves as secondary results of earlier specific causes.

The history ot this disease in man and domestic animals points to the fact that these gum borders, liable as they are to degenerations, are intimately dependent on the functional health of possibly every gland, membrane and nerve centre in the body, and on the quality and quantity of the body juices, on cachexiæ and diatheses.

I have been accused by some of my dearest friends and most kindly critics of not making positive statements in some former essays, and here I wish to announce that this Riggs' disease—let us call it so out of compliment to this early systematic student of the trouble—has as many remote, predisposing internal or systemic causes as has catarrh and the eczemas, and offers as wide a field for the specialists' treatment—this specialist having the broadest possible general medical training—as does chronic nasal catarrh by the rhinologist and the eczemas by the dermatologist.

Let me be positive again. The far-away predisposing cause of Riggs' disease is frequently a neurosis. The neurasthenic, professional and business man ; the stay-at-home wife, overburdened with the monotony of her existence ; the epileptic and the paralytic exhibit this disease as a sequence of neurosis, and, as the psycho-physiologist might show, as a result of abnormal emotions.

Now, the dentist who attacks the local manifestations is acting in a perfectly reasonable and logical manner, and does much good by his delicate surgery and topical treatment, but the remote causes remain alert and ready for attack at any time that local opportunities offer. When we can treat and cure gland disturbance and rebuild nerve tissue, and strike the hydra-headed gout and put all the blood-elaborating and waste-eliminating organs within the pale of physiology, with all that this means in the struggle for an existence, we may prevent this disease from occurrence and recurrence, and we are no farther away from this millenium of therapeutics than are our brilliant and worthy confreres in other departments of medicine.

The sensitive nerve terminals and reflex motor responses, the capillary circulation, the leucocytes congregated at the point of distress, are all sensitive to local impressions and susceptible to intelligent medication. And it may be proper to assert, just here, that in the treatment of constitutional phases, or more remote special organs like the nervous system, or the intestinal tract, or other diseases like syphilis or tuberculosis, or concurrent catarrhal manifestations that consultations with the family physician or other specialists should be insisted upon for the benefit of the patient, that the broadest measures in therapeutics may be adopted and harmoniously carried out by the combined knowledge, methods and training which are the result only of special study. I have no sympathy with the specialist who is sufficient unto himself, whether his practice is confined to diseases of the nervous system, to surgery, to internal medicine or to general practice.

Local treatment has in many cases proven successful in catarrh, in eczema and in this disease. Extraction cures caries and it cures alveolar abscess and pyorrhea alveolaris, but the aim of the dental surgeon of to-day is to save teeth.

May I be permitted to suggest that as in the local treatment of this disease of the gums the most delicate and intelligent surgery is required and a persistent patience not necessary in more brilliant efforts like the removal of a tumor or the mastoid operation, so-called, and as time and skill are the business assets of the operator, all old ideas about compensation that have grown up between dentist and patient must be done away with. We are placed in the curious position of being able at any moment, by surgical elimination, to effect a radical cure of the disease, and yet our earnest desire and the demands of cultured patients are all for the saving of the teeth. We have become imbued with the doctrine that the dentist's highest aim is to save to usefulness and beauty the natural teeth. Our lives are spent in this effort—in fighting the diseases which threaten the destruction of these organs. We fight caries, pulpitis, alveolaritis and Riggs' disease—not a very long list, but chronic, progressive and complicated in character. This special Riggs' disease is the most destructive in its effects upon the patient and upon his teeth, and requires longer, more persistent and more frequent operations than any other, not excepting caries. The recognition of these facts is important to dentist and patient.

The adjustment of compensation to the dentist for his efforts in battling with Riggs' disease must be upon the plane of the other specialists in medicine who treat the eye, the ear, the nose or the throat. If we can not rise to this plane, (and this may be why many dentists offer no hope of palliation by treatment) then we should retire to the older platform and recommend extraction and plates.

The general health of the patient afflicted with Riggs' disease demands intelligent and perhaps prolonged treatment and constant watchfulness, followed by persistent hygienic attention, or the radical cure by extraction; and we, as conservators of the health of our patients and in the interests of preventive medicine, should plainly state the case to those who come to us for advice.

### DISCUSSION.

Dr. Junius E. Cravens, of Indianapolis, complimented the essayist for the skillful manner in which he had treated this subject, especially since he had studiously avoided antagonizing any of the pet theories as to its nature and treatment. He believed that Riggs' disease had its origin in some local lesion of the gum border or periosteum. It may be possible that the frequency of this disease at this date may be due to the use of wooden tooth-picks, dental operations requiring the use of clamps, separators, unclean scalers, etc. Too often these and similar agencies are the cause of injuries which give rise to infectious diseases and destructive inflammatory consequences.

Destructive irritants in the form of salivary calculus without and intoxicating waste products from within prevent the natural processes of repair and create a condition of chronic inflammation. Repair processes may be active, but the destructive processes predominate and we have gradual wasting of the peridental structures. A serous secretion at first may become fibrous, hemorrhagic or purulent, as the essayist states, and unless local remedies are applied the destruction of the tissues is inevitable. That the disease has systemic relations is patent, but it is the local manifestations that largely concern the dentist and which will generally yield to intelligent local treatment.

DR. J. TAFT, of Cincinnati: Heretofore this disease has not had the serious attention of dentists that it deserved. Much of the old writing is valueless except for history; only a few wrote worthily. Chapin A. Harris and H. H. Hayden were early writers upon this subject and much that they wrote is of interest and value to-day.

Dr. Riggs was the first to write upon this subject from the operative standpoint; he ignored its systemic complications and also largely its medicinal treatment. How well he succeeded in combating the disease by local instrumentation is well known. His work was so well received by the profession that the disease is often referred to as Riggs' disease.

DR. C. N. PIERCE, of Philadelphia, has been the most painstaking writer on the systemic relations of this disease. He found it often associated with rheumatic and gouty diseases, and many other prominent workers have written at length upon this subject—Talbot, Black, Kirk.

The essayist has fairly presented its status to-day. A more exhaustive study of the oral tissues and their secretions will no doubt help to clear up the etiology of this disease. Its treatment by local interference in the early stages is successful. It should be kept continually under the observation when once diagnosed, and in cases where its heredity may be suspected such patients should be warned of its probable approach.

Parents should be told to caution their children, so that they may procure treatment when it will be of the greatest service.

